# Epigenetics of aging and disease: a brief overview

**DOI:** 10.1007/s40520-019-01430-0

**Published:** 2019-12-06

**Authors:** Christina Pagiatakis, Elettra Musolino, Rosalba Gornati, Giovanni Bernardini, Roberto Papait

**Affiliations:** 1 Department of Biotechnology and Life Sciences, University of Insubria, Varese, Italy; 2 Department of Cardiovascular Medicine, Humanitas Research Hospital, Rozzano, MI Italy; 3 Genetic and Biomedical Research Institute, National Research Council of Italy, Rozzano, 20089 Milan Italy

**Keywords:** Epigenetics, Aging, Cardiovascular disease, Cancer

## Abstract

Aging is an important risk factor for several human diseases such as cancer, cardiovascular disease and neurodegenerative disorders, resulting from a combination of genetic and environmental factors (e.g., diet, smoking, obesity and stress), which, at molecular level, cause changes in gene expression underlying the decline of physiological function. Epigenetics, which include mechanisms regulating gene expression independently of changes to DNA sequence, regulate gene expression by modulating the structure of chromatin or by regulating the binding of transcriptional machinery to DNA. Several studies showed that an impairment of epigenetic mechanisms promotes alteration of gene expression underlying several aging-related diseases. Alteration of these mechanisms is also linked with changes of gene expression that occurs during aging processes of different tissues. In this review, we will outline the potential role of epigenetics in the onset of two age-related pathologies, cancer and cardiovascular diseases.

## Introduction

Aging is associated with a progressive decline of numerous physiological processes, which is accompanied by an increase of risk for development of severe diseases such as cancer, cardiovascular disease, senile dementias and type-II diabetes in the elderly. Interestingly, the incidence of many cancers increases after 50 years of age, and diagnoses of heart failure (HF) increases from the age of 60 years, with most patients being over 70 years old. This, coupled with the fact that the average life span has increased, is resulting in aging having a considerable importance at both economic and social levels [1]. Despite this, the mechanisms that link aging with the onset of aging-related diseases remain largely unknown.

The aging process of an organism results from a combination of stochastic events including both genetic and environmental factors (e.g., diet, smoking, obesity and stress), which, at the molecular level, cause changes in gene expression underlying the decline of physiological function. For example, aging of the brain is accompanied by changes of expression of genes encoding proteins involved in inflammatory and stress responses and neuropeptide metabolism, while the elderly heart has an altered transcription profile that contributes to impairment of heart function [2].

Furthermore, aging-related diseases are consequences of an impairment of gene expression. For example, cancer is caused by alteration of gene expression that leads to cellular acquisition of neoplastic characteristics (e.g., proliferation that is not under the control of the organism, loss of differentiation and ability to metastasize to distal tissues). These changes can be triggered by genetic mutations or stochastic events that cause changes in the gene expression programs [3]. In general, the subjection of DNA, RNA, and proteins to chemical alterations throughout the lifespan impairs both their structure and function. The consequences of nucleic acid and protein damage during aging have been linked to functional deterioration of cells and organs, inevitably leading to disease [4]. While heart failure is accompanied by two pathological processes, cardiac hypertrophy and cardiac fibrosis, the underlying source results from alterations of gene expression. Cardiac hypertrophy is accompanied by an increase of expression of fetal cardiac genes (e.g., *Nppa*, *Nppb*, *Myh7* and *Skeletal Alpha*-*Actin*) and repression of adult genes (e.g., *Myh6*), while cardiac fibrosis is a result of an increase of expression of genes encoding proteins of the extracellular matrix (e.g., collagens), processes for which the specific underlying mechanisms still remain unknown [5].

Several studies over the last decade have strongly implicated epigenetic mechanisms in the (dys)regulation of the gene expression changes regulating several aging-related diseases such as cancer and heart failure, and in promoting the alteration of gene expression responsible for the aging process of different tissues. Therefore, alteration of the epigenetic mechanisms occurring during aging renders cells more prone to the transcriptional changes responsible for aging-related diseases. The impairment of gene expression, and the multifactorial nature of epigenetic changes throughout the aging process and disease onset render their mechanisms of action enigmatic: it is, thus, of utmost importance to elucidate these mechanisms to understand the origins of age-related disease. In this short review, we will discuss the possible role of epigenetics in regulating the onset of two aging-related diseases, cancer and cardiovascular disease.

## A brief overview of epigenetics

**Fig. 1:**
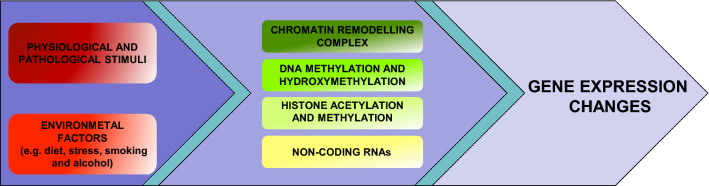
Overview of the epigenetic mechanisms that mediate the effects of physiological and pathological stimuli and environmental factors on gene expression

Epigenetics refers to all the mechanisms regulating gene expression independent of the DNA sequence, which can be grouped into four main processes: ATP-dependent chromatin-remodeling complexes, DNA and histone modifications, and non-coding RNAs. These processes regulate gene expression by modulating the structure of chromatin or by regulating the binding of transcriptional machinery to DNA. One important feature of these mechanisms is that they can be modulated by a myriad of factors including physiological and pathological stimuli, as well as by environmental factors such as diet, stress, physical activity, working habits, smoking and alcohol consumption (Fig. 1) [6–8].

ATP-dependent chromatin-remodeling complexes are multi-protein complexes that regulate gene expression by modifying the nucleosome organization of DNA using energy derived from ATP hydrolysis. Members of these families function as transcriptional activators by promoting the formation of an open and accessible chromatin structure, allowing the recruitment of several proteins involved in transcription. For example, SWI/SNF complexes promote the formation of this structure through a mechanism involving sliding nucleosomes, evicting the H2A/H2B dimers or removing the histone octamers from DNA. Other chromatin-remodeling factors cause gene silencing by organizing the nucleosomes on DNA in a manner that results in the chromatin structure taking a highly compacted form, and thus preventing accessibility to transcription factors [9].

DNA modifications are covalent modifications of DNA bases: the most commonly studied modifications are methylation and hydroxymethylation of cytosine bases. DNA methylation occurs predominantly on the cytosine within CG-dinucleotide-rich genomic regions. These genomic regions are called CpG islands and are found in the majority of promoters in both the human and mouse genomes. The methylation of cytosine promotes transcriptional repression by acting as docking sites of methyl-CpG binding domain (MBD) proteins, a family of proteins that promotes the formation of silent chromatin. In mammals, the DNA methylation pattern is established and maintained by three DNA methyltransferase enzymes (DNMTs): DNMT3A and DNMT3B are essential for de novo DNA methylation during development [10], while DNMT1 is required for maintaining methylation patterns during cell division. Moreover, DNA hydroxymethylation is the product of hydroxylation of 5-mC (5-methylcytosine) catalyzed by ten–eleven translocation (TET) enzymes. High levels of 5-hmC (5-hydroxymethylcytosine) in promoter and enhancer regions are linked with high levels of transcription [11].

Histone modifications are covalent post-translational modifications, which include acetylation, methylation, phosphorylation, ubiquitylation and sumoylation. Among these, the best studied are acetylation and methylation. Acetylation occurs on lysine residues present in the tails of histones, allowing transcription factor accessibility as a result of neutralization of the positive charge of histone tails [7]. The level of acetylation of chromatin depends on the activity of two classes of enzymes, histone acetyltransferases, which catalyze the transfer of an acetyl group from acetyl-coenzyme A to lysine residues present in the tails of histones, and histone deacetylases which remove this acetyl group [7]. Histone methylation is another important epigenetic marker, whose effects on transcription depend on the specific position and degree of lysine and arginine methylation on histone tails. Briefly, histone methylation associated with transcription activation occurs as di- or tri-methylation of histone H3 at lysines 4, 36 and 79 (H3K4, H3K36 and H3K79, respectively) and mono-methylated H3K9 and H4K20; whereas, transcriptional repression is characteristic of tri-methylation of H3K9, H3K27, H4K20 and di-methylation of H3K9, which are involved in gene silencing through formation of facultative and constitutive heterochromatic regions [6, 7]. Just like histone acetylation, methylation is a dynamic process resulting from the activity of two classes of enzymes: histone methyltransferases, which catalyze the transfer of a methyl group from *S*-adenosyl-methionine to lysine or arginine residues of histones, and histone demethylases, which catalyze the demethylation of histone tails.

The last class of epigenetic mechanisms involves noncoding RNAs (ncRNAs), which include a variety of RNAs that are not translated into proteins. Non-coding RNAs are classified based on their length: short and long. The class of short ncRNAs includes molecules of RNA shorter than 200 nucleotide such as PIWI-interacting RNAs, small interfering RNAs (siRNAs), and microRNAs (miRNAs). In contrast, long ncRNAs (lncRNAs) includes molecules of RNA longer than 200 nucleotides. NcRNAs regulate the expression of proteins at the transcriptional and translational levels. The lack of species conservation of lncRNA renders its study more difficult, although their temporal and spatial expression could be key to understanding the regulation of chromatin structure, recruitment of transcriptional machinery and gene expression. Conversely, microRNAs inhibit the expression of genes by binding to the 3′-UTR (untranslated region) of their target mRNAs, resulting in a degradation of the target mRNA and a subsequent inhibition protein translation [12].

Over the last few decades, the involvement of epigenetic mechanisms has been implicated in several aging-related diseases. In this review, we will focus our attention on two key pathologies, cancer and cardiovascular disease.

## Ageing and epigenetics in pathologies

### Aging, cancer and epigenetics

Cancer is a disease that results from accumulation of genetic and epigenetic alterations, and for several cancers, the most important risk factor is age [13]. Perturbations to the genome as a result of changes to cellular environment, inflammation, decrease immune function and accumulation of DNA damage, result in malignant transformation and carcinogenesis [14].

**Fig. 2:**
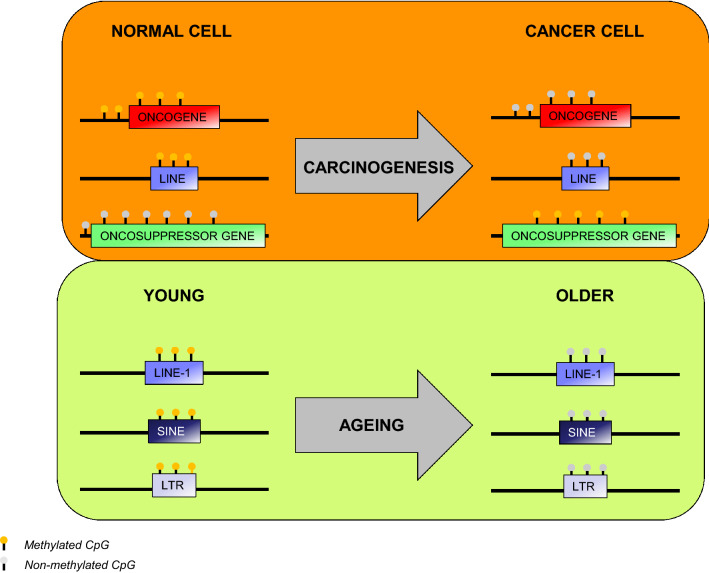
Summary of DNA methylation changes in carcinogenesis and aging

In an effort to elucidate the role of epigenetic regulation in cancer, several groups over the last few decades have focused their attention on the canonical epigenetic mechanisms that are perturbed during cancer. Dysregulation of DNA methylation is one of the most common epigenetic perturbations in cancer. Loss of DNA methylation at specific regulatory and repetitive elements such as Alu elements (Alu) and long interspersed element-1 (LINE1) has been associated with an increase in genomic instability, making chromosomal arrangements that lead to tumor formation more likely [15, 16]. Conversely, it has been shown that in certain types of tumors, the hypermethylation of CpG islands in the promoter regions of tumor-suppressor genes has been associated with development of cancer (Fig. 2) [17].

Moreover, aberrant activity of the enzymes catalyzing histone modifications contributes to carcinogenesis [18]. For example, EZH2 (enhancer of zeste homologue 2), the catalytic subunit of PRC2, which mediates the deposition of the H3K27me3 repressive mark, has been implicated in several cancer types, whereby its expression is increased in prostate cancer, breast cancer, lymphomas and glioblastomas [19–21]. Conversely, the histone lysine demethylase JMJD2C, which catalyzes the demethylation of H3K9, has been associated with breast and esophageal cancers, de-repressing genes involved in these pathologies [22, 23]. Moreover, the dynamic process of histone acetylation, which is generally associated with transcriptional activation, is involved in a variety of cancer types; the balance of the activity of histone acetylases (HATs) and histone deacetylases (HDACs) is necessary in the maintenance of cellular homeostasis by regulating chromatin conformation and transcription states [24]. In fact, aberrant regulation of several HATs has been found to be responsible for gene expression changes underlying carcinogenesis. For example, the Gcn5 HAT has been implicated in breast cancer due to dysregulation of Wnt signaling [25]. Furthermore, the altered expression of the MOZ gene and its paralog MORF, which encode for two histone acetyltransferases that act as co-transcriptional activators, contributes to the development of myeloid leukemia [26]. Finally, p300 and CBP HATs have been identified as key tumor-suppressor proteins, and their dysregulation was described in many cancer types [27]. Conversely, HDACs are also involved in several cancers, whereby increase in their expression potentiates tumorigenesis in breast, prostate and colorectal tissues as a result of hypoacetylation of several gene loci [24]. Interestingly, deacetylation of non-histone proteins such as p53 and YY1 transcriptional repressors and the STAT3 transcriptional activator has been implicated in carcinogenesis in many cell types [28].

Studies regarding the role of epigenetics in aging have been greatly focused on the role of DNA methylation in this physiological process. Global levels and genomic distribution of 5-methylcytosine change across the genome and at specific loci during aging. Interestingly, DNA methylation has been evaluated as a biomarker for determining the age of cells and tissues, and loci-specific methylation patterns can be very telling as to the age of various tissues. Exposure to ROS, which increases DNA damage, inflammation and the activity and function of DNMTs, has been shown to be correlated with the deposition of 5mC and 5hmc and an increase in mutation frequency [29]. Furthermore, studies have shown that damage occurring to DNMT genes during aging causes a significant decrease in global methylation levels in repetitive elements such as LTRs (long terminal repeat), SINEs (short interspersed nuclear element) and LINE-1 (long interspersed element-1) (Fig. 2) [30–34]. Studies have also shown that specific loci containing hypomethylated enhancers are linked to genes whose expression is regulated during aging, and the presence of hypermethylation was shown to be enriched at CpG islands, which is associated with carcinogenesis onset [35].

Dysregulation of genes during aging is also linked to an alteration of histone methylation: in murine models, changes in H3K4me3 (activating) and H3K27me3 (repressing) levels have been directly linked to lifespan. Therefore, the DNA and histone modification changes occurring during aging could contribute to the definition of an epigenome more inclined to acquire epigenetic changes responsible for tumor onset.

Finally, several non-coding RNAs have been described in regulating cell mechanisms associated with aging, including proliferation, differentiation, apoptosis and senescence, which in turn, have been implicated in potentiation of carcinogenesis [36]. Some examples of long non-coding RNAs that have been studied in this context are MALAT1 (metastasis-related lung adenocarcinoma transcript 1), SALNR and HOTAIR (HOX transcript antisense RNA), which are necessary in regulating cellular processes such as promotion of tumor cell proliferation, invasion, metastasis, drug resistance, and angiogenesis, thus playing a key role cancer progression as a result of aging [37].

### Aging and cardiovascular diseases and epigenetics

Cardiovascular disease (CVD), leading to heart failure, and subsequently death, is the major cause of morbidity and mortality worldwide [38]. There are several risk factors linked to the development of CVD, including hypertension, diabetes and obesity [39]. However, one of the main risk factors of CVD is age, with its prevalence, including atherosclerosis, stroke and myocardial infarction, increasing in the elderly [40]. Aging patients present with several functional changes to the heart, including diastolic and systolic dysfunction, arrhythmias, and atrial fibrillation, to name a few [41]. As with all aging pathophysiologies, the high prevalence of CVD in the aged population has been linked to inflammation, oxidative stress, production of ROS, apoptosis, myocardial deterioration and degeneration [42]. Furthermore, the inflammatory response results in cardiac remodeling, with significant changes to the extracellular matrix, and the presence of proinflammatory and inflammatory markers (IL-6, TNFα, CRP) [42]. Cardiac remodeling subsequently leads to the development of cardiac hypertrophy and fibrosis, an effect that is pronounced in aged hearts, leading to impairment of cardiac function [43]. Development of fibrosis has been considered to be the starting point of several pathophysiologies, which is initiated by an aberrant inflammatory response, and results in structural and functional deterioration of many organs [44].

Of great importance to the proper functioning of the heart is the mitochondria, which is necessary for the metabolic activity of the heart and the production of ATP [45]. Cardiac dysfunction in the elderly has been correlated with mitochondrial dysfunction as a result of oxidative stress and production of ROS [41], factors which play a detrimental role to the capacity of mitochondrial respiration [46]. Studies have shown that the development of atherosclerosis in the aged population has been linked to lipid oxidation as a result of mitochondrial dysfunction [47]. Oxidative stress also contributes to impaired calcium signaling, which is required for maintenance of the sarcoplasmic reticulum and muscle contraction [48].

Epigenetic dysregulation has been associated with several cardiovascular pathologies and cardiovascular aging: some of the key epigenetic mechanisms include DNA methylation and hydroxymethylation, chromatin remodeling, aberrant changes in histone modifications, and dysregulation of non-coding RNAs [49, 50]. DNA methylation, which has been shown to be impacted by several environmental factors, is a key player in the genetic regulation of genes necessary for cardiac homeostasis, regulating various cell processes required for proper cardiac function. DNA methylation patterns are maintained by DNMT1, whereas de novo DNA methylation is mediated by DNMT3A and DNMT3B [50]. Furthermore, DNA methylation can be reversed through oxidation of 5-methylcytosine to 5-hydroxymethylcytosine (5-hmC) by the TET (ten–eleven translocation) proteins [50]. Global changes in DNA methylation have been shown to change during aging, and have been correlated with the onset of several cardiovascular pathologies [51].

Chronic inflammation, a key factor of initiation of cardiomyocyte stress and initiation of pathological onset, has been associated with unbalanced lipid levels occurring during atherosclerosis [49]. Studies in patients with dyslipidemia have revealed a subset of genes including CPT1A, with differential methylation profiles in control versus affected individuals. CPT1A is necessary for the maintenance of healthy lipid profiles by regulating mitochondrial function, and aberrant changes in its methylation status have been associated with lipoprotein and triglycerides in the blood [52]. Hypermethylation of the promoter region of ATP-binding cassette A1 (ABCA1), which is required for cholesterol transfer from blood to high-density lipoprotein particles, was found in patients with familial hypercholesterolemia [53]. Furthermore, epigenetic changes in several genes including *RELA, NOS3, KLF4* and *APOE* have been linked to progression of atherosclerosis [54]. An important mechanism in the progression of atherosclerosis is smooth muscle cell (SMC) proliferation; under quiescent conditions, SMCs maintain a differentiated phenotype, aiding in vascular tone. Under pathological conditions of stress or inflammation, SMCs become more proliferative and migratory, contributing to the formation of atherosclerotic lesions [55]. This dysregulation has been associated with changes in methylation of several genes, including the Estrogen Receptor gene (ESR-a) [56], collagen type XV alpha 1 (COL15A1) [57] and transforming growth factor, beta receptor III (TGFBR3) [58].

**Fig. 3:**
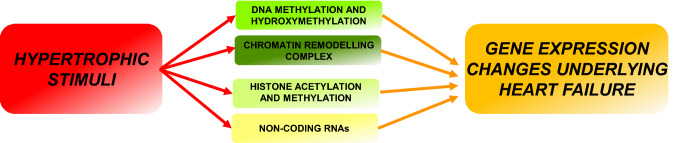
The classes of epigenetic mechanism that define the transcriptional changes of cardiac hypertrophy

As with the onset of cardiac hypertrophy, in which cardiac remodeling results in a gene expression shift mimicking that of the fetal stage [5], the same changes in DNA methylation patterns in aging have been observed [50]. Furthermore, changes in 5-hmC patterns in hypertrophic hearts also resemble the neonatal stage: these changes in methylation and hydroxymethylation have been associated with the regulation of key cardiac genes such as *MYH7, MYOCD, SRF* and *KLF4* [50], although such changes during cardiac aging still remain elusive. Although the study of histone modifications and chromatin remodeling has been a topic of great interest in the study of cardiovascular disease, and there have been great advances in understanding the specific mechanisms regulating cardiac homeostasis, many aspects in the context of aging are still unclear. The histone methyltransferase G9a/Ehmt2 has been implicated in the maintenance of cardiac homeostasis by interacting with EZH2, and MEF2C (myocyte-specific enhancer factor 2C) to maintain the heterochromatin needed for repressing fetal gene expression in the adult heart [5]. Conversely, after onset of pathological hypertrophy, expression of miR-217 inhibits G9a, resulting in a reduction of H3K9me2, thus de-repressing the fetal gene program and resulting in pathological remodeling of cardiomyocytes [59]. Moreover, the study of histone deacetylases (HDACs) in the regulation of cardiac hypertrophy has been a topic of great interest over the last few decades, and several studies have shown that this class of enzymes is necessary in the maintenance of cardiomyocyte homeostasis [60, 61]. Finally, the chromatin remodeling complex, BAF (SWI/SNF), has been shown to be critical to cardiomyocyte function, and its dysregulation has been implicated in loss of cardiovascular function and onset of pathology by regulating several cardiac-specific genes including *MYH6*. Furthermore, studies have revealed that in injured adult mouse hearts, there is an alteration of binding of BAF to genomic DNA, thus altering gene expression profiles (Fig. 3) [62].

Non-coding RNAs have been of great interest in the regulation of cardiomyocyte homeostasis and onset of cardiovascular disease. Over the last few years, many studies have outlined the importance of several ncRNAs that are altered in cardiac and age-related cardiac pathologies. For example, microRNAs miR-21, miR-22, miR-34a and the miR17-92 cluster are significantly altered in cardiac aging, regulating proteins important in fibroblast–myofibroblast transition [63], cardiac fibroblast senescence [64], cardiac contraction [65] and collagen synthesis [66], respectively.

Finally, of great importance, and of great interest over the last few years, is whether RNA methylation could be subject to epigenetic modifications. Although several chemical modifications can be found on RNA, RNA methylation (N6-methyladenosine, m6A) is the most abundant modification in eukaryotic messenger RNAs. This chemical modification has been found to be present in intronic regions of messenger RNA, transfer RNA, ribosomal RNA, and noncoding RNAs [67]. RNA methylation is a dynamic process and is mediated by writers, readers and erasers, just like DNA methylation, and the role of these proteins has been gaining much interest in several biological contexts and pathophysiologies [67]: the role of this chemical modification of RNA in pathogenesis and aging, however, remains largely unknown. Recent studies have revealed that RNA methylation is necessary for the maintenance of cardiac homeostasis via a mechanism driven by METTL3, a protein responsible for RNA methylation. This study revealed that in vivo overexpression of METTL3 increased m6A and resulted in hypertrophic growth of the heart, and furthermore, that cardiomyocyte-specific knockout of METTL3 resulted in a decrease of cardiac function after pressure overload and during aging [68]. Further, elucidation of the role of METTL3 and the role of RNA methylation in aging is necessary to understand the mechanisms by which perturbations in these modifications may play a role in aging and the onset of pathogenesis. Conversely, FTO (fat mass and obesity-associated protein), an m6A eraser, has been shown to decrease in mammalian failing hearts thus reducing the contractile function of cardiomyocytes [69]. Although its role in aging has not yet been elucidated, the understanding of the balance between RNA methylation writers and erasers will be necessary in uncovering the mechanisms perturbed during aging-induced pathologies.

Taken together, these (and other) studies have unveiled several epigenetic mechanisms regulating cardiac homeostasis and revealed how dysregulation of these pathways can lead to onset of cardiovascular pathologies. Many of epigenetic mechanisms involved in CVD are also implicated in aging of several tissues. Despite this, the role of epigenetics in cardiovascular aging and in the onset of cardiovascular disease in the elderly still remains to be elucidated.

## Conclusions and future directions

Over the last decade, our understanding and knowledge of the epigenetic mechanisms involved in cellular homeostasis and disease have greatly increased. Mechanisms involving histone post-translational modifications, chromatin remodeling and non-coding RNA have revealed multi-layered mechanisms regulating the homeostatic state of several cell type (cardiomyocytes, fibroblasts, immune cells, etc.); mechanisms which when dysregulated result in the onset of cancer and cardiac pathologies. Although epigenomic dysregulation in CVD alone is still not fully understood, the role of epigenetics in aging and age-related diseases remains even more elusive. Much progress is still needed to understand changes in the epigenetic landscape of the aged cardiomyocyte (both physiological and pathological), the pathways of which are critical for the maintenance of cardiomyocyte homeostasis, and which are the key factors (environmental or other) that influence chromatin architecture and govern gene expression changes that promote disease.

Furthermore, despite decades of study of carcinogenesis, the mechanisms (both genetic and epigenetic) governing the aberrant gene expression changes, cellular identity and responsiveness to cellular and environmental cues, ultimately resulting in cancer, still remain unknown. Studies have revealed that targeting epigenetic mechanisms could play a profound and possibly ubiquitous role in abolishing tumorigenesis. As with other age-related pathologies, there are several unanswered questions with respect to correlating mechanisms of carcinogenesis and aging.

Moreover, recent work has shown, using RNA sequencing of whole blood in healthy aged subjects, that there is a small subset of genes that is characterized by changes in expression and splicing and a reduction in genetic regulation, late in life. Quantifying allele-specific gene expression, alternative splicing and genetic regulation, this study showed that there is a strong correlation between genetic effects and the increase in allelic imbalance with age, which is furthermore correlated with increasing environmental variance [70]. Recent studies have also emphasized the importance of the role of maintenance of heterochromatin, and the dysregulation of chromatin structure during the aging process. Indeed, the heterochromatin protein HP1 has been suggested to be required for longevity, and its dysregulation has been implicated in progeroid syndromes [71]. Using genome-wide approaches in aged animal models will be key to understanding changes in the epigenetic landscape of aged cells and tissues to better correlate pathology-specific gene expression changes. Exploiting new technologies, such as assay for transposase accessible chromatin sequencing (ATAC-seq), in human aging models, to elucidate changes in chromatin accessibility in cardiac and tissues during and post-disease onset, will be critical in understanding changes at the epi-transcriptome level [72]. Correlation of these changes in chromatin accessibility with changes in gene expression profiles can be further linked using RNA-sequencing, Hi-C analysis, and ChIP-sequencing of aged cardiac tissue to specifically map changes in genomic and epigenetic domains.

Since epigenetic changes have been shown to be reversible, manipulation of epigenomic pathways and epigenetic enzymes using CRISPR/Cas9 technology or targeted pharmacological inhibition could prove to be crucial in reversing epigenetic aberrations that are a hallmark of aging. Currently, there are several epigenetic drugs that are either commercially available, or under clinical phase trials [73]. These drugs target an array of epigenetic enzymes including histone modifying enzymes and DNA methyltransferases [74, 75]. Exploiting these drugs for pathologies beyond cancer will be critical for the understanding mechanisms and onset of several age-related diseases. The main challenge will be to test these drugs in aged animal models, and elucidate their function and role in a tissue-specific manner, avoiding off-target effects.

Finally, single-cell transcriptome analysis will be crucial to uncovering epigenomic changes within various cellular and sub-cellular populations to further shed light on the correlation of (epi)transcriptomic changes in aged and diseased tissue. Single-cell RNA sequencing of a murine cardiac pressure-overload HF model revealed that induction of HF leads to an intricate immune activation, whereby a myriad of cells, including neutrophils, B cells, NK cells and mast cells are activated [76]. Using this approach, and correlation of aberrant or acute immune responses with disease onset during aging can further shed light onto the underlying mechanisms of the failing heart. To this end, a recent study in muscle stem cells revealed that there are context-dependent alterations of DNA methylation with age. Using single-cell transcriptomic analysis, this study showed that there is a degradation of functional transcriptional networks correlated with an increase in heterogeneity between the epigenome and the transcriptome during aging [77]. Using these approaches in a context- and tissue-specific manner can reveal the subtle, yet critical changes underlying pathophysiologies associated with aging.
